# Co-delivery of dimeric camptothecin and chlorin e6 via polypeptide-based micelles for chemo-photodynamic synergistic therapy

**DOI:** 10.1186/s13020-023-00817-6

**Published:** 2023-10-13

**Authors:** Zhaopei Guo, Ka Hong Wong, Enze Li, Xingzhi Zhou, Di Jiang, Jiebing Gao, Meiwan Chen

**Affiliations:** 1 State Key Laboratory of Quality Research in Chinese Medicine, Institute of Chinese Medical Sciences, University of Macau, Macau, SAR China; 2 Department of Radiology, The Fifth Affiliated Hospital, Sun Yat-sen University, Zhuhai, 519000 China

**Keywords:** Chemo-photodynamic therapy, Combination cancer therapy, Polypeptide, Camptothecin

## Abstract

**Background:**

The integration of photodynamic therapy with a chemical drug-delivery system has displayed great potential in enhancing anticancer therapy. However, the solubility and non-specific biodistribution of both chemotherapeutic agents and photosensitizers continue to pose challenges that hinder their clinical applications.

**Method:**

A polypeptide-based nanoscale drug delivery system was fabricated to address the prementioned issues. An amphiphilic polymer was formed by conjugating the photosensitizer chlorin e6 (Ce6) onto a polypeptide poly-(L-lysine)-b-polyphenylalanine (PKF) for encapsulating the model drug dimeric camptothecin (DCPT), and the nanoparticles (PCD) with high drug loading efficiency were further modified with acid-sensitive polyethylene glycol (PEG) to yield the drug delivery sytem (PPCD).

**Results:**

The DCPT and Ce6 encapsulation efficiency were analyzed as 99% and 73.5%, respectively. In phosphate-buffered saline (PBS) solution at a pH of 7.4, the PEG shell improved the stability of micelles and shielded their positive charge while in the acidic tumor microenvironment, the pH-sensitive PEG layer was removed to expose the cationic nanoparticles, thus facilitating the cellular uptake of PPCD micelles. Benefiting from the enhanced cellular internalization, the amount of intracellular reactive oxygen species (ROS) treated with PCD and PPCD micelles were obviously increased. Furthermore, the enhanced anti-cancer efficacy prompted by PPCD micelles was validated through cellular and animal study.

**Conclusion:**

This study presents a promising method to promote the solubility and biodistribution of both chemotherapeutic agent and photosensitizer, thereby facilitating the further application of chemo-photodynamic cancer therapy.

**Graphical Abstract:**

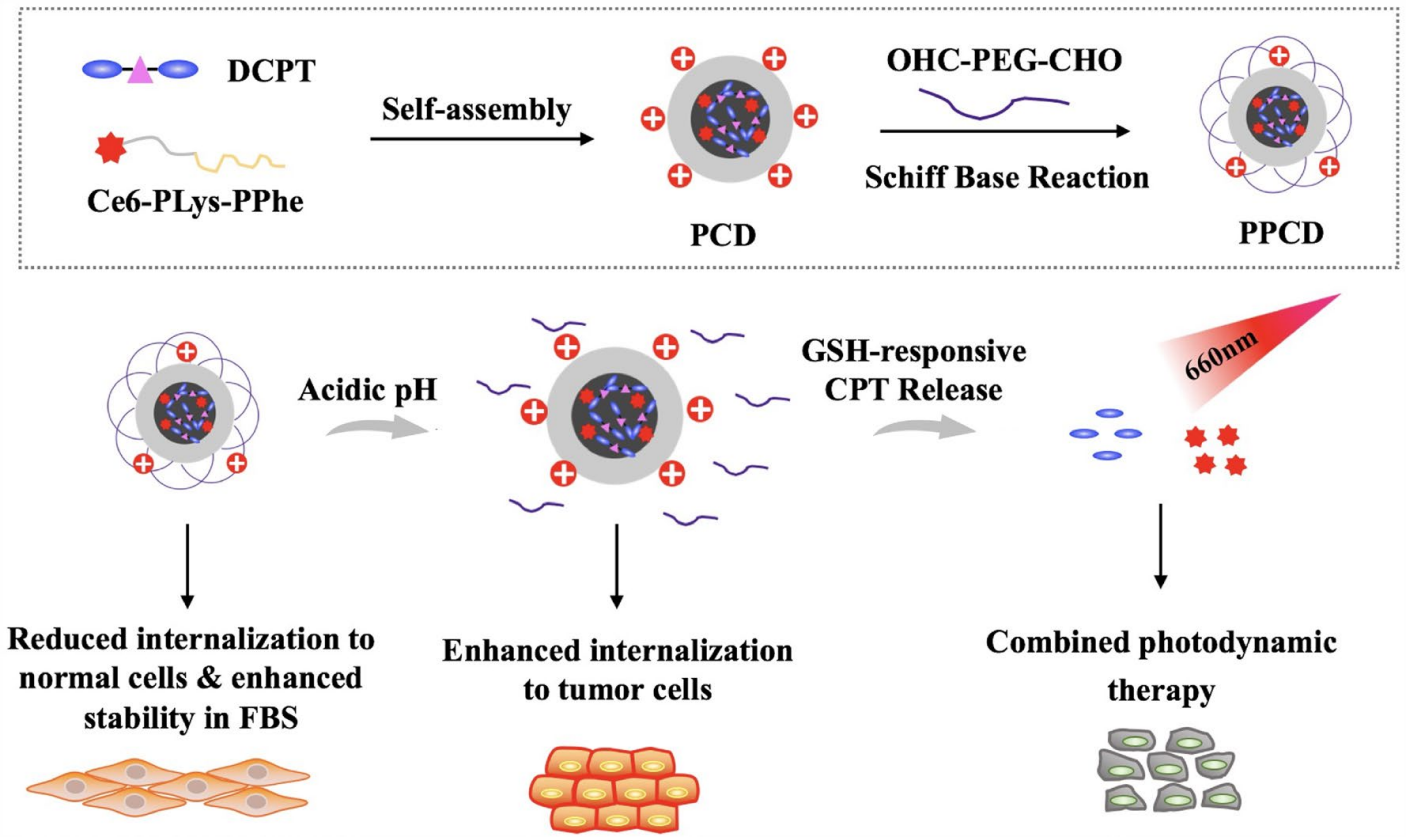

**Supplementary Information:**

The online version contains supplementary material available at 10.1186/s13020-023-00817-6.

## Introduction

Lung cancer is a major contributor to cancer-related deaths, which brings about over one million fatalities per year and is deemed as a main public health issue [1, 2]. Up till now, surgery, chemotherapy and radiotherapy are the main therapeutic regimens for lung cancer treatment [3, 4]. However, the applicaions of these therapies are restricted by assorted side effects linked to the general toxicity of frequently used therapeutics agents [5]. Combination therapy is emerging as an alternative promising approach as it contributes to favorable therapeutic efficiency when compared to the respective monotherapy [6]. Photodynamic therapy (PDT) is a local treatment with minimal invasiveness, negligible side effects, decreased long-term morbidity and without resistance acquiring mechanisms. There are three core components of PDT including photosensitizers (PSs), light and oxygen, which are individually non-toxic. When the three factors are integrated, PSs will be initiated to occur a series of photochemical reactions and yield singlet oxygen with great reactivity, leading to cell death [7, 8]. It is reported that combining PDT with chemotherapy can boost PDT efficiency and lower the adverse effects of chemotherapy agents. For example, combining gemcitabine and the PS chlorin e6 (Ce6) significantly inhibited the viabiliity of 4T1 cells under light irradiation and also decreased the adverse effects of free gemcitabine [9]. Nonetheless, the potential application of PDT is hampered by some barriers, including the poor solubility and low tumor accumulation of PSs [10]. In this regard, exploiting nanomedicine-based delivery systems for PS and chemotherapeutic drug delivery is of great value for cancer treatment.

Camptothecin (CPT) has been employed as second-line drugs for patients with first-line chemotherapy treatment failure in cancer therapy because they showed no significant cross-resistance to first-line agents like cisplatin and paclitaxel [11, 12]. In addition, CPT-based nano drug delivery systems for combination therapy exhibited promising therapeutic effects [13, 14]. In particular, Yue et al. fabricated a thioketal linker-modified CPT and then loaded this drug with Ce6 into a PEG-coated upconversion nanoparticle. Upon light irradiation, Ce6 was activated to generate ROS, which significantly damaged NCI-H460 lung cancer cells. Moreover, the induction of ROS cleaved the thioketal linker, leading to the release of CPT for chemotherapy, thus enabling the implementation of a chemo-photodynamic combined therapy [13]. However, simply loading PS and chemotherapeutic drug simultaneously often accompanied with poor drug loading, and the multi-dose caused potential inflammatory response and systemic toxicity. To overcome this issue, introducing one of the chemotherapeutic drug or PS to polymer chains can greatly improve the drug loading efficiency [15, 16]. For instance, Gao et al. prepared a doxorubin-conjugated macromolecular prodrug for curcumin encapsulation, greatly enhancing both drug loading efficacy [16].

Herein, a co-delivery system for dimeric CPT/Ce6 (DCPT/Ce6) was constructed to realize chemo-photodynamic combination cancer therapy. In this nanostructure, the PS Ce6 was conjugated onto the backbone of the polymer chain to increase the drug loading efficiency while CPT was conjugated with a disulfide linker to yield the DCPT for encapsulation efficiency improvement by increasing the intermolecular interaction [17–19]. Ce6 possesses high singlet oxygen yield and great potential in PDT for deep tumors due to its long absorption wavelength [20, 21]. Meanwhile, CPT was released from the micelles for chemotherapy under redox environemt in cancer cells, thus realizing chemo-photodynamic therapy against cancer. To prepare the system, PKF polypeptide polymer was firstly synthesized and then Ce6 was conjugated onto the PKF polymer to create PKF-Ce6. PKF-Ce6/DCPT (PCD) micelles were obtained through a dialysis method, followed by CHO-PEG-CHO modification to yield the micelles (PEG-PKF-Ce6/DCPT, PPCD). Bonding with pH-responsive PEG can effectively reduce the surface charge of amphiphilic cationic polymer. Also, the PEG layer can also reduce non-specific adsorption and increase long-term circulation in the body, thereby improving passive targeting of the micelles. Upon delivery to the acidic tumor microenvironment, the pH-sensitive PEG layer can be detached to expose the cationic micelles, increasing the binding of nanoparticles to cell membranes, and improving endocytosis. This co-loaded system prepared with high drug loading efficiency possesses not only the merits of pH-sensitive PEG detachment but also demonstrates the ability to suppress A549 tumors through the synergy of DCPT-based chemotherapy and Ce6-based PDT. This offers a novel appropach for potential chemo-photodynamic cancer therapy in the future.

## Materials and methods

### Materials

CPT was procured from Dalian Meilun Biology Technology Co., Ltd (Liaoning, China) and used to synthesize DCPT as per our previous report without any modifications [18, 22]. Phenylalanine (Phe), 1-ethyl-3-(3-dimethylaminopropyl)carbodiimide hydrochloride (EDC), N-ε-Cbz-L-lysine, and 1-hydroxy-2,5-pyrrolidinedione (NHS) were bought from GL Biochem Ltd (Shanghai, China). Ce6 was provided by Frontier Scientific (Utah, USA). Dimethyl sulfoxide (DMSO) was purchased from Aladdin (Shanghai, China). N, N-dimethylformamide (DMF) was purchased from Damao (Tianjin, China). Hydrogen bromide in acetic acid (33 wt%) and bis(trichloromethyl) carbonate were supplied by J & K Scientific Ltd (Beijing, China). Thiazolyl blue tetrazolium bromide (MTT) was purchased from Amresco (Solon, Ohio, USA). PEG_6000_ were obtained from JenKem Technology Company (Beijing, China). DAPI were purchased from Beyotime Biotechnology (Jiangsu, China).

### Synthesis and characterization of PKF-Ce6 conjugate

First, N-carboxyanhydride of phenylalanine (Phe-NCA) and N-ε-Cbz-L-lysine (Lys(Z)-NCA) were synthesized as previously reported [23]. Then, polylysine-polyphenylalanine (PKF) was synthesized as following steps [18, 24]. Briefly, a solution of 3.27 mmol of Lys(Z)-NCA in 15 mL of anhydrous DMF was subjected to ring-opening polymerization (ROP) by adding 0.11 mmol of hexylamine. After 72 h of reaction at 30 °C under nitrogen protection, followed by precipitation, PLys (Z) was obtained and further used to trigger the ROP of Phe-NCA at room temperature to synthesize PLys(Z)-PPhe. Then, purified PKF was attained by deprotecting the carbobenzoxy groups of PLys(Z)-PPhe using trifluoroacetic acid (TFA) and HBr solution, followed by precipitation in pre-cooled ether, dialysis against water, and freeze-dry under vacuum. The synthesis of PKF was confirmed by ^1^ H NMR spectroscopy (Bruker, USA).

In addition, Ce6 was further grafted onto the PKF polypeptide through the following process: First, Ce6 (18 mg), EDC and NHS (20 mg for each) were added in 3 mL of DMSO and allowed to react for 30 min, thus initiating the carboxyl group of Ce6. Later, the mixture was added into PKF (in DMSO) and reacted for 1 d. Then the crude product was transferred into a dialysis tube containing DMSO for another 24 h. An UV-Vis spectrophotometer (HACH, USA) was employed to characterize the synthesized PKF-Ce6 polymer.

### Formulation and characterization of PCD and PPCD micelles

PCD and PPCD micelles were fabricated using dialysis method. In brief, PKF-Ce6 (at an equivalent Ce6 mass of 100 µg) and 500 µg of DCPT dissolved in DMSO were dripped into 2 mL of water and allowed to stir for 2 h. PCD was yielded after dialysis (with a MWCO 3500 Da) and centrifugation (4700 r/min, 5 min). Furthermore, appropriate amounts of α,ω-diformyl poly(ethylene glycol) (OHC-PEG-CHO, about 4–6 times of PKF), which was synthesized according to reported procedures [25], were added to PCD for 30 min of reaction, yielding the PPCD micelles. The particle sizes and zeta potential of PCD and PPCD micelles were assessed using a Nano-ZS Zetasizer instrument (Malvern, UK). The encapsulation efficiency of DCPT and Ce6 were measured by UV-Vis spectrophotometer at 380 or 660 nm, respectively. Then, the morphology of PCD and PPCD was examined using a transmission electron microscope (FEI Tecnai). To assess the pH-sensitivity of the PEG detachment, 0.5 mL of both PCD and PPCD were combined with 0.5 mL of PBS (pH 7.4 or 6.5) and incubated for 15 min. Changes in the zeta potential of the micelles were examined. For in vitro release of DCPT from PPCD, dialysis (MWCO: 3500 Da) of 1 ml of PPCD against 20 ml of PBS with different pH and GSH conditions was performed. The release of DCPT was monitored by drawing 1 ml of sample at various time points. The fluorescence intensity of DCPT in the sample was read by using a SpectraMax microplate reader (λ_ex_: 370 nm and λ_em_: 435 nm).

### Cell culture

The human lung adenocarcinoma cell line A549 and PC-9 cells were offered by American Type Culture Collection (MD, USA). Cells were incubated in DMEM supplemented with 10% (v/v) fetal bovine serum (FBS), 1% penicillin, and 1% streptomycin under 5% CO_2_ at 37 °C.

### Cellular uptake

A549 and PC-9 cells were planted in 12-well plates with a number of 1.0 × 10^5^ cells per well. Then, DMEM at pH 7.4 or 6.5, containing Ce6, PCD and PPCD micelles (all at an equivalent Ce6 concentration of 1 µM) were added. After 1, 4, or 8 h, the cells were rinsed and harvested to mearsure the fluorescence intensity of Ce6 by a BD FACS Canto™ flow cytometer (NJ, USA).

Additionally, the cellular uptake of micelles was observed using the Leica SP8 CLSM system (Wetzlar, Germany). Firstly, A549 and PC-9 cells were planted with a number of 1.5 × 10^5^ cells per dish in individual confocal petri dish. Subsequently, the cells were exposed to DMEM (pH 7.4 or 6.5) mixed with Ce6, PCD and PPCD micelles (equivalent Ce6 concentration of 2 mM). After 8 h-incubation, cells were fixed and stained with DAPI (5 µg/mL) and observed under a CLSM.

### ROS Measurement

The ROS generating ability of PCD and PPCD after laser irradiation were detected by 1,3-DPBF reactive oxygen probes (Merck, USA). Breifly, 20 µg of DPBF and 2 µg of Ce6 (in PCD or PPCD) were added to 996 l of DMF solution and exposed to a laser (660 nm, 100 mW/cm^2^). The decolarization of DPBF after light irradiation was assessed using a UV spectrophotometer at a wavelength of 415 nm. In addition, intracellular ROS production was evaluated through the application of DCFH-DA ROS assay kit (Beyotime Biotechnology, Jiangsu, China). Firstly, A549 and PC-9 cells were seeded in 12-well plates with a number of 1.5 × 10^5^ cells per well. Subsequently, free CPT, free DCPT, free (CPT + Ce6), free (DCPT + Ce6), PCD and PPCD micelles (equivalent Ce6 1 µM) were introduced and incubated for 8 h. The intracellular ROS was then stained with DCFH-DA (20 µM) for a duration of 20 min. After that, each well was subjected to laser irradiation (660 nm, 100 mW/cm^2^, 10 min) or left untreated. The fluorescence signals were determined by a flow cytometer.

### Cell viability assay

A549 and PC-9 cells were placed in 96-well plates with a number of 8 × 10^3^ cells per well. Once the cells had adhered, DMEM containing various CPT concentrations of free CPT, free DCPT, free (CPT + Ce6), free (DCPT + Ce6), PCD and PPCD micelles were added for cell culture at both pH 7.4 and pH 6.5 with an incubation period of 24 h. Then, medium was substituted with MTT-containing medium (1 mg/mL) and incubated for 4 h. Following this, the medium in each well was discarded and substituted with 100 µL of DMSO to dissolve the formazan crystals. Finally, the absorbance of each well was read by microplate reader at 570 nm. The cell viability was calculated based on at least three independent experiments.

Furthermore, the chemo-photodynamic combination therapeutic effects against A549 and PC-9 cells were carried out. Similarly, cells were subjected to varying concentrations of Ce6, including free Ce6, free (CPT + Ce6), free (DCPT + Ce6), PCD and PPCD micelles, under both pH 7.4 and pH 6.5 conditions. After an incubation period of 8 h, the treated cells were subjected to a laser (660 nm, 100 mW/cm^2^) for 10 min, followed by an additional 16 h-incubation. At last, the relative cell viabilities were determined using MTT assay and compared to the control group.

### *Visually observation of combination effects*

With the aim to directly visualize the combination therapy efficacy, A549 and PC-9 cells were planted into 6-well plates with a number of 2 × 10^5^ cells per well. Medium containing CPT, DCPT, as well as medium supplemented with free Ce6, free (CPT + Ce6), free (DCPT + Ce6), PCD or PPCD micelles (equivalent Ce6 1 µM) were introduced to replace culture medium. Following an incubation period of 8 h, the subsequent five groups underwent a laser irradiation (660 nm, 100 mW/cm^2^) for 10 min. After an additional 0.5 h-incubation, the cells were rinsed with buffer solution and then stained with Calcein-AM/PI double stain kit (Yeason, Shanghai, China) for viable and non-viable cells labelling. The corresponding fluorescence images were taken by a GE Healthcare Life Science IN Cell Analyzer 2000 (MA, USA).

### Cell apoptosis

The micelle-induced cell apoptosis was further invesigated by a Cell Signaling Technology Annexin V-FITC kit (MA, USA). Briefly, A549 and PC-9 cells were seeded in 12-well plates and exposed to various formulations including free CPT, free DCPT, free Ce6, free (CPT + Ce6), free (DCPT + Ce6), PCD and PPCD micelles. After an 8-h incubation, the latter five groups were subjected to laser irradiation (660 nm, 100 mW/cm^2^) for 10 min, followed by an additional 12 h-incubation. Sequently, cells were collected and gently suspended in 96 µL of binding buffer, followed by staining with 1 µL of Annexin-FITC together with 12.5 µL of PI. Finally, the apoptosis of cells was investigated using flow cytometry.

### Animals

BALB/C mice (female, 6–7 weeks) and BALB/c nude mice (male, 5 weeks) were provided by Gunagdong Medical Laboratory Animal Center (Guangzhou, China). For tumor model establishment, a suspension of 5 × 10^6^ A549 cells in 100 µL of PBS was inoculated into the right flank of each nude mouse.

### Pharmacokinetics study

Female BALB/C mice were allocated into 3 groups (n = 3) randomly. Prior to the study, mice were fasted with water for overnight. An equivalent dose of 5 mg per kg body weight of DCPT was administered by different routes (PCD and PPCD: intravenously injection; DCPT: intraperitoneally injection). 10 µl of blood samples were obtained from the tail vein at various time intervals, which was diluted to 50 µL with PBS for measurement. Pharmacokinetic study of Ce6 was performed using another 3 groups of female BALB/C mice. Equivalent 1 mg per kg body weight Ce6 dose was administered by different routes (PCD and PPCD: intravenously injection; Ce6: intraperitoneally injection). Fluorescence intensity of DCPT and Ce6 in blood sample was recorded by using a SpectraMax microplate reader (DCPT: λ_ex_ = 370 nm and λ_em_ = 435 nm; Ce6: λ_ex_ = 402 nm and λ_em_ = 652 nm).

### Assessment of therapeutic efficacy in vivo

Once the tumor grew to a size of approximately 100 mm^3^, the mice were categorized into 5 treatment groups (n = 5): PBS, PCD, PPCD (intravenous administration), DCPT, Ce6 (intraperitoneal administration). The administration does was every 3 days, with total 5 times injection (DCPT dose: 5 mg per kg body weight). Following a 12-h interval post-administration, the tumor site of each mouse was performed laser irradiation (660 nm, 100 mW/cm^2^) for a duration of 15 min. The tumor size was assessed using a vernier caliper, and body weight was monitored every alternate day. The tumors were calculated according to the formular: V = 0.5 length x width^2^. After completion of the entire therapeutic regimen, the mice were euthanized to dissect the main organs and tumor tissues for histopathological evaluation. After fixing with 10% (W/V) formaldehyde, embedding in paraffin, slicing, and stainging using hematoxylin and eosin, the 3-µm slices were examined using an Olympus BX43 microscope (Tokyo, Japan).

### Statistical analysis

All data are expressed as the mean ± SD of independent measurements unless explicitly stated otherwise. Statistical significance was indicated when *p* < 0.05 (denoted by *), < 0.01 (denoted by **) and < 0.001 (denoted by ***).

## Results and discussion

### Synthesis and characterization of PKF-Ce6 conjugate

**Fig. 1:**
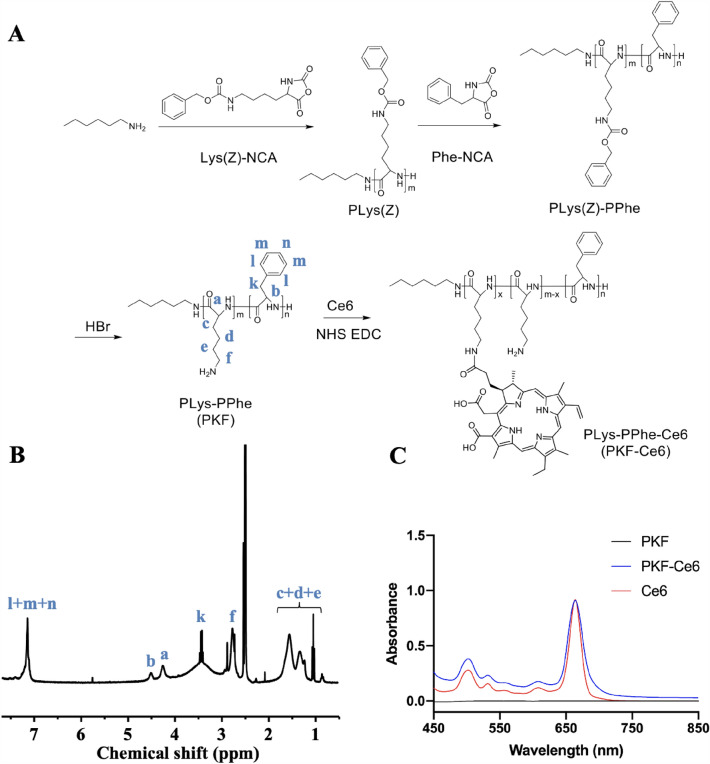
Illustration of PKF synthesis **A** and corresponding ^1^ H-NMR spectra **B**; **C** UV absorbance of Ce6, PKF and PKF-Ce6 polymer (equivalent Ce6 concentration: 20 µg/mL)

Figure 1 A shown the synthesis process of PKF and the ^1^ H-NMR spectra revealed the successful manufacture (Fig. 1B). PKF-Ce6 conjugate was obtained by the amide formation between the amine group on PKF polypeptide and the carboxyl group on Ce6. Furthermore, the occurrence of 660 nm-absorption peak in PKF-Ce6 indicated the successful grafting of Ce6 onto PKF polypeptides and there was no UV absorption shift after conjugation (Fig. 1C). Thus, the mass percentage of Ce6 contained in the PKF-Ce6 was determined to be 23% by using UV spectroscopy.

### Preparation and characterization of PCD and PPCD micelles

**Fig. 2:**
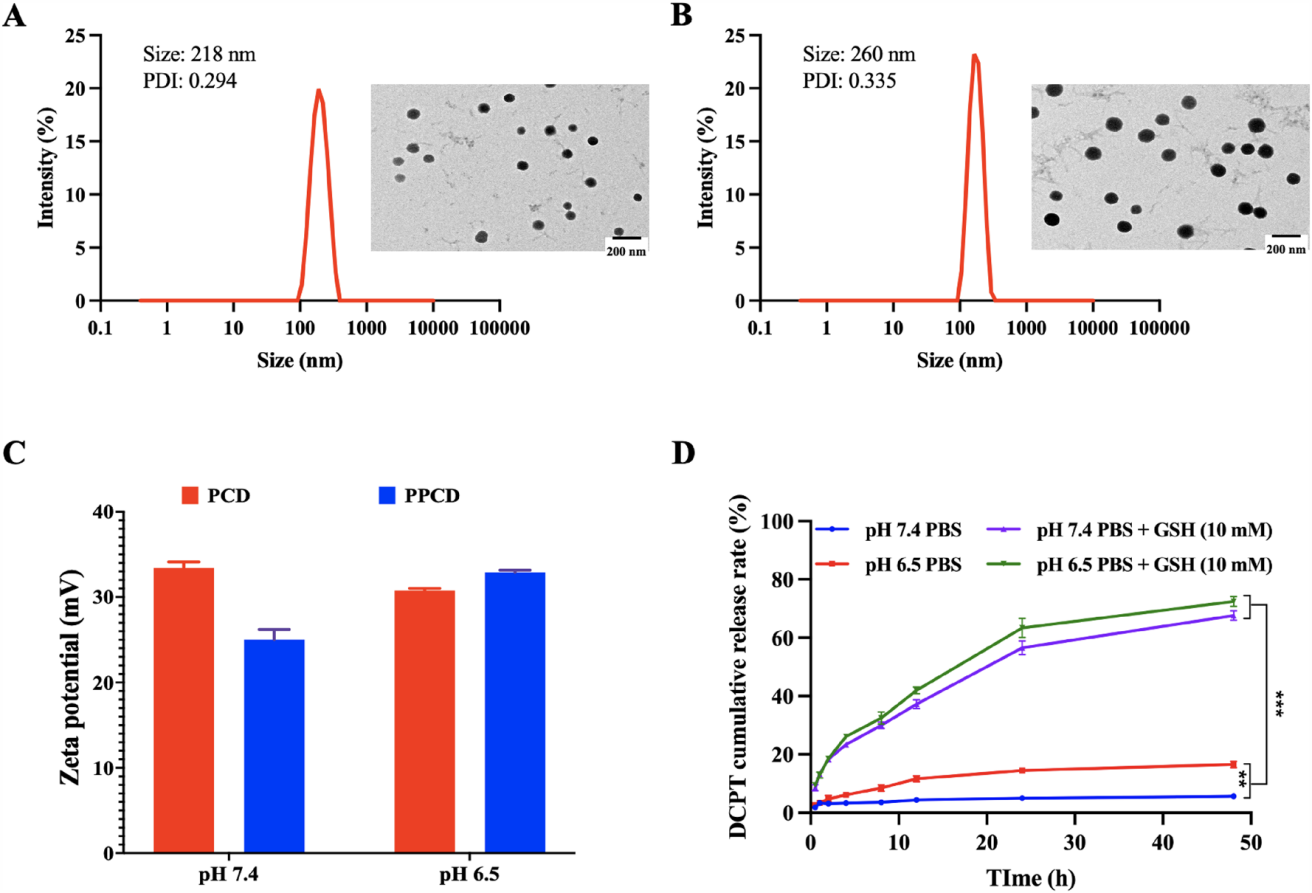
Characterization of PCD and PPCD micelles. Particle size and respective images of PCD **A** and PPCD **B** taken by TEM; **C** Zeta potential of PCD and PPCD in PBS at various pH; **D** Release pattern of DCPT from PPCD under different conditions in vitro

The optimal mass ratio of Ce6 and DCPT was determined to be 1:5 (Additional file 1: Table S1). This ratio was applied to the physical mixture of free drugs for further cell experiments. The micelles were spherical particles with particles sizes of 215.4 ± 3.9 nm and 257.0 ± 5.3 nm, respectively (Fig. 2A, B), the particle sizes were slightly increased as compared to the blank micelle (PKF-Ce6 micelle) after drug loading (Additional file 1: Fig. S1). In the meantime, the encapsulation efficiency of Ce6 and CPT in PPCD micellar formulation was measured as 73.5% and 99%. The PEG attachment on the micelles was verified by treating PCD and PPCD against acidic environment. Notably, the zeta potential of PPCD was was reduced compared to that of PCD micelles in both PBS solution with a pH of 7.4 and pure water. The reduction can be attributed to the shielding effect of CHO-PEG-CHO. When the zeta potentials of PPCD and PCD were investigated in PBS solution at a pH of 6.5, the values of zeta potential of PPCD increased to the parallel levels of PCD, indicating the detachment of PEG under acidic environment (Fig. 2C). The release manners of DCPT from PPCD were assessed in PBS solution with pH values of 7.4 or 6.5, both with and without the presence of 10 mM of GSH. Only small amount DCPT released from the micelles without the addition of GSH. When 10 mM of GSH was present, DCPT exhibited rapid release regardless of pH environment, indicating that the disulfide-crosslinking strategy can maintain the composition of nanoparticles, and achieving controlled release in redox environment [26].

### Cellular uptake

**Fig. 3:**
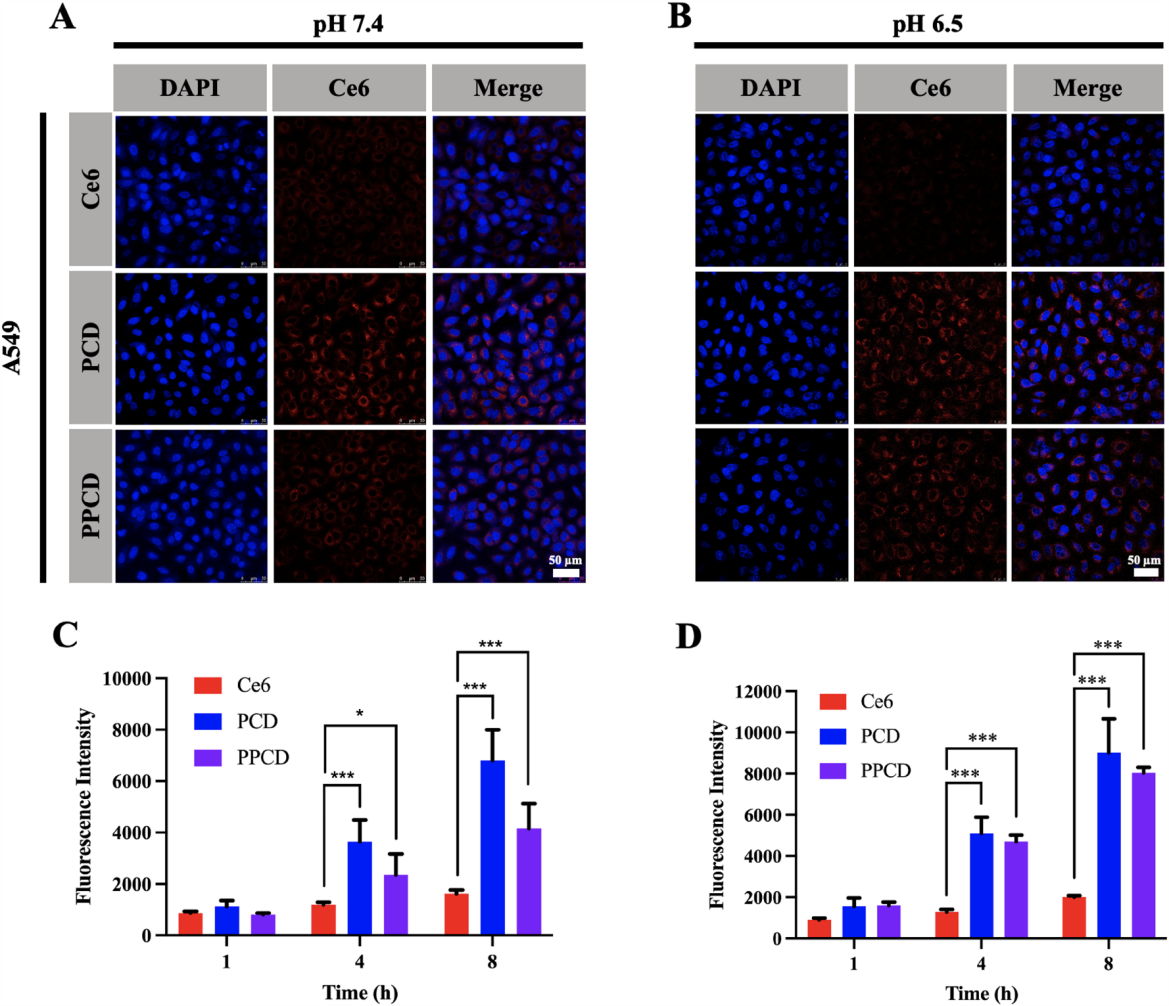
Cellular uptake of PCD and PPCD in A549 cells at pH 7.4 **A** or pH 6.5 **B**. Representative images taken using fluorescent microscopy (Scale bar: 50 μm). Fluorescence intensity of the free Ce6, PCD and PPCD in A549 cells at pH 7.4 **C** or pH 6.5 **D** measured by flow cytometry

**Fig. 4:**
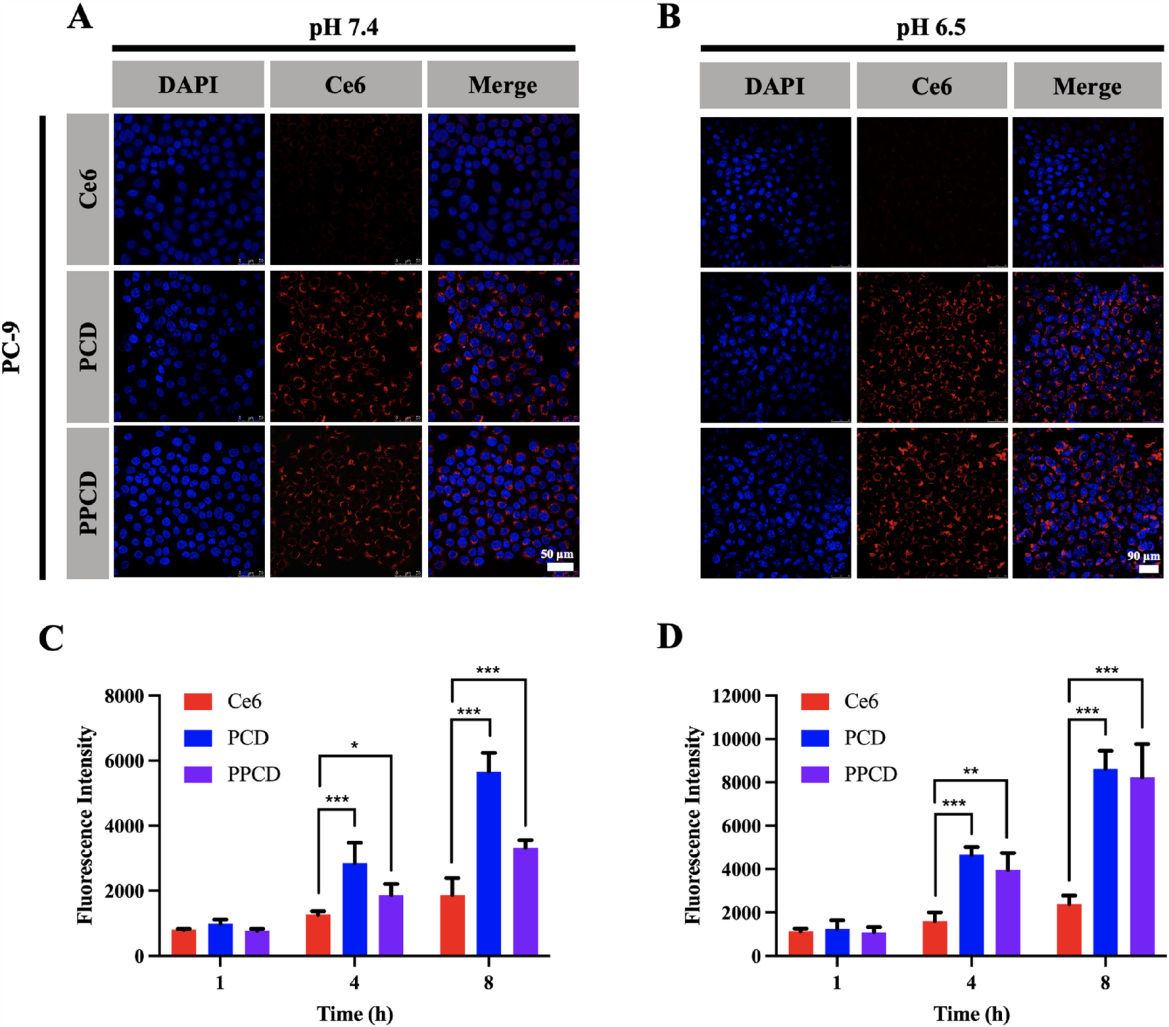
Cellular uptake of PCD and PPCD in PC-9 cells at pH 7.4 **A** or pH 6.5 **B**. Representative images taken using fluorescent microscopy. Fluorescence intensity of the free Ce6, PCD and PPCD uptake in PC-9 cells at pH 7.4 **C** or pH 6.5 **D** measured by flow cytometry

As illustrated in Figs. 3 and 4, A549 cells and PC-9 cells treated with PCD and PPCD micelles exhibited superior fluorescence intensity compared to those treated with free Ce6. This observation suggests that the drug-loaded nanoparticles significantly enhance the cell internalization in comparison to the free drugs. Particularly, in the medium with pH 7.4, cells exposed to PPCD micelles exhibited a decreased fluorescence intensity compared to the cells treated with PCD. However, under pH 6.5 environment, the fluorescence intensity of PPCD-treated cells showed an obvious enhancement, while there was no improvement in the PCD-treated groups. The observed outcomes could potentially be attributed to the grafting of PEG onto the surface of PCD micelle. Briefly, in a normal physiological environment, the PEG layer enveloped the nanoparticles surface to reduce the endocytosis of non-target cells by decreasing the interaction between the cell membrane and nanoparticles [27]. Once the nanoparticles reach the slightly acidic tumor environment, the pH-responsive PEG layer is removed, thereby increasing the endocytosis efficiency of the positive-charged nanoparticle by the tumor cell. This observation was in accordance with the results in the zeta potential changes of PPCD uder acid conditions (Fig. 2C), further comfirming the pH-sensitive PEG detachment.

### ROS Measurement

**Fig. 5:**
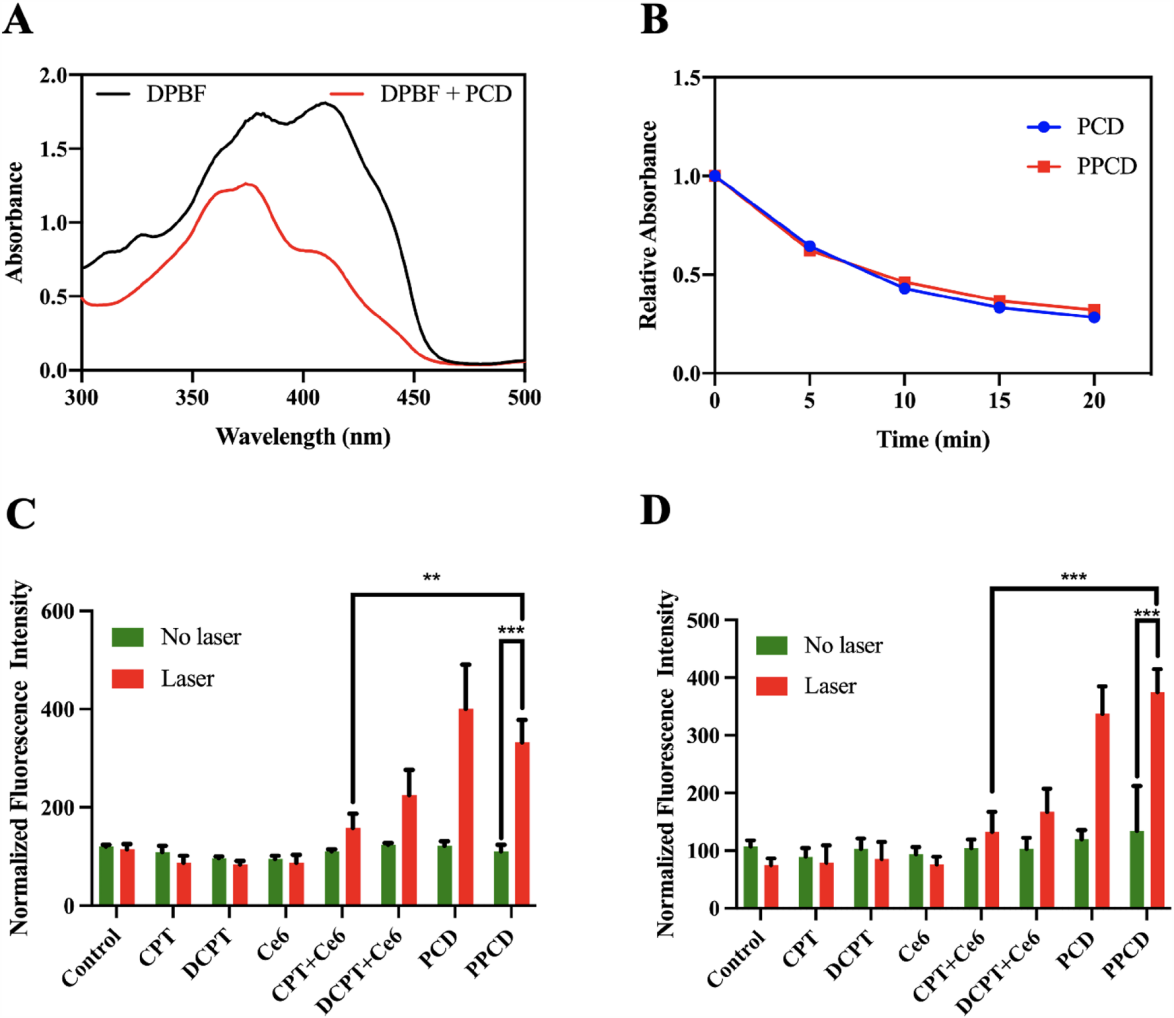
**A** Detection of ROS produced by PCD after light irradiation using DPBF probe. **B** Comparison of the ROS producing ability of PCD and PPCD using DPBF probe. Quantitative measurement of intracellular ROS in A549 cells **C** and PC-9cells **D** using flow cytometry. Control was untreated cells

The ability of PCD and PPCD micelles after light irradiation to produce ROS was firstly investigated by using DPBF as a probe (Fig. 5A). As shown in Fig. 5B, both PCD and PPCD produced ROS in a time-dependent manner. And there was no significant difference when comparing the fading process of the probe, indicating that the modification of PEG corona did not alter the ROS generating ability of PCD. Then, the intracellular ROS inducing ability of PCD and PPCD was studied in A549 cells and PC-9 cells. From Fig. 5C, D, the fluorescence intensity of A549 and PC-9 cells incubated with free CPT, DCPT and Ce6 with or without laser illumination remained notably low. This could potentially be attributed to the limited cellular internalization of these compounds. And ROS generated in free (CPT + Ce6) and free (DCPT + Ce6) groups slightly increased. Conversely, when cells were treated with PCD and PPCD micelles plus laser irradiation, ROS production was significantly enhanced, which was owing to the enhanced cellular internalization. This observation aligns with the findings in cellular uptake.

### Cell viability assay

**Fig. 6:**
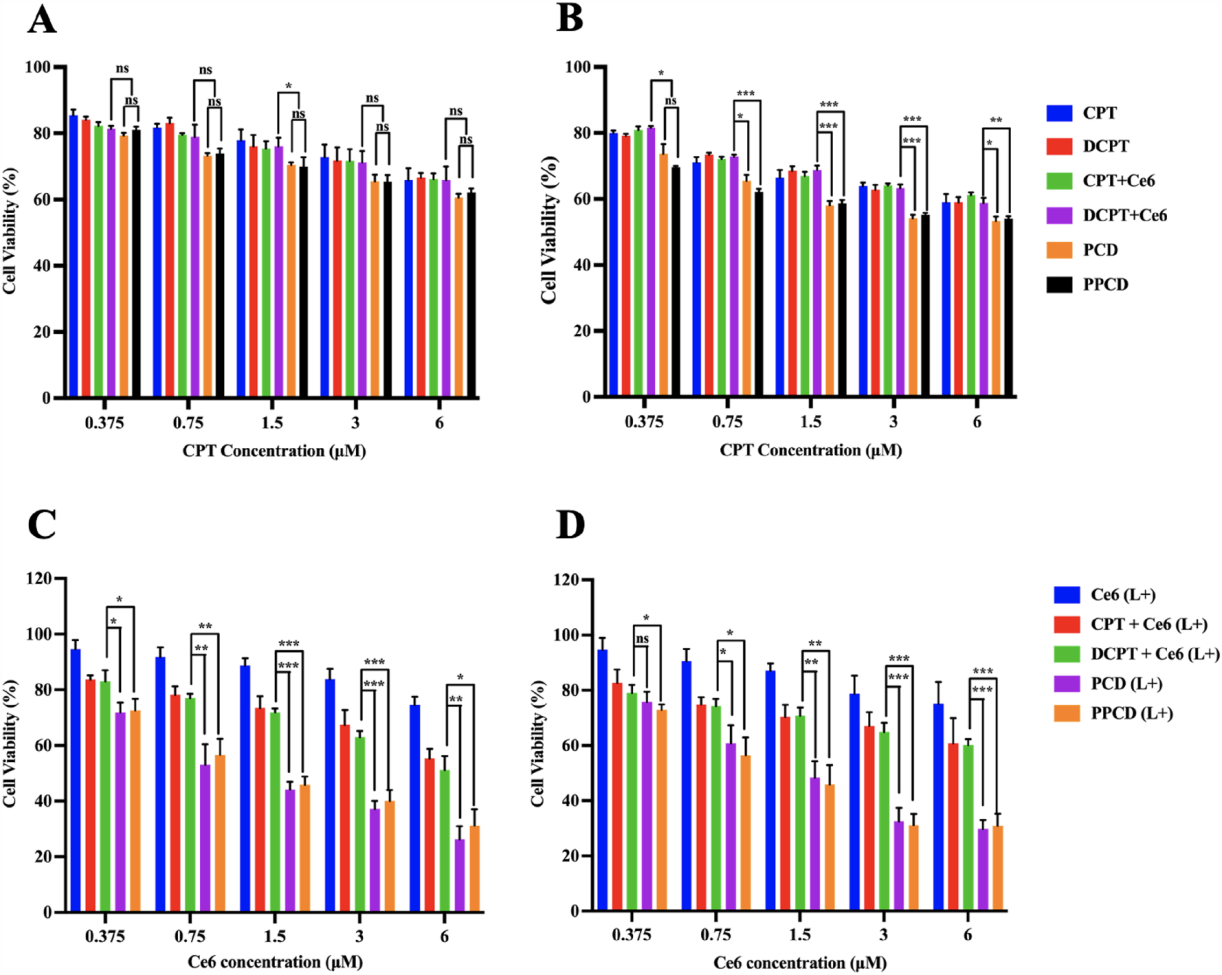
In vitro cytotoxicity evaluation of PCD and PPCD. Chemotherapy effects on A549 **A** and PC-9 **B** cells without laser illumination; Chemo-photodynamic effects on A549 **C** and PC-9 **D** cells with laser irradiation (L+)

The cell inhibition effects of chemotherapy against A549 and PC-9 cells were evaluated and compared through MTT assay. As show in Fig. 6A, B, all treatment groups without laser irradiation showed weak concentration-dependent cytotoxicity, and PCD as well as PPCD micelles showed the strongest inhibitory effects, which may be contributed to the enhanced cellular uptake. Subsequently, chemo-photodynamic anticancer efficacy of micelles was investigated upon laser irradiation. As expected, PCD and PPCD micelles resulted in obviously improved antitumor effects, showing the benefits of chemo-photodynamic combinational effects. Basically, the effectiveness of PDT in cancer therapy is related to the tumor type and penetration ability of light source. In clinic, sufficiently small size of optical fibers or LED light arrays under CT guidance can be employed to deliver light for PDT in lung tumors [28]. Therefore, it is possible to apply the developed system in clinic in future.

### Visual observation of combination effects

**Fig. 7:**
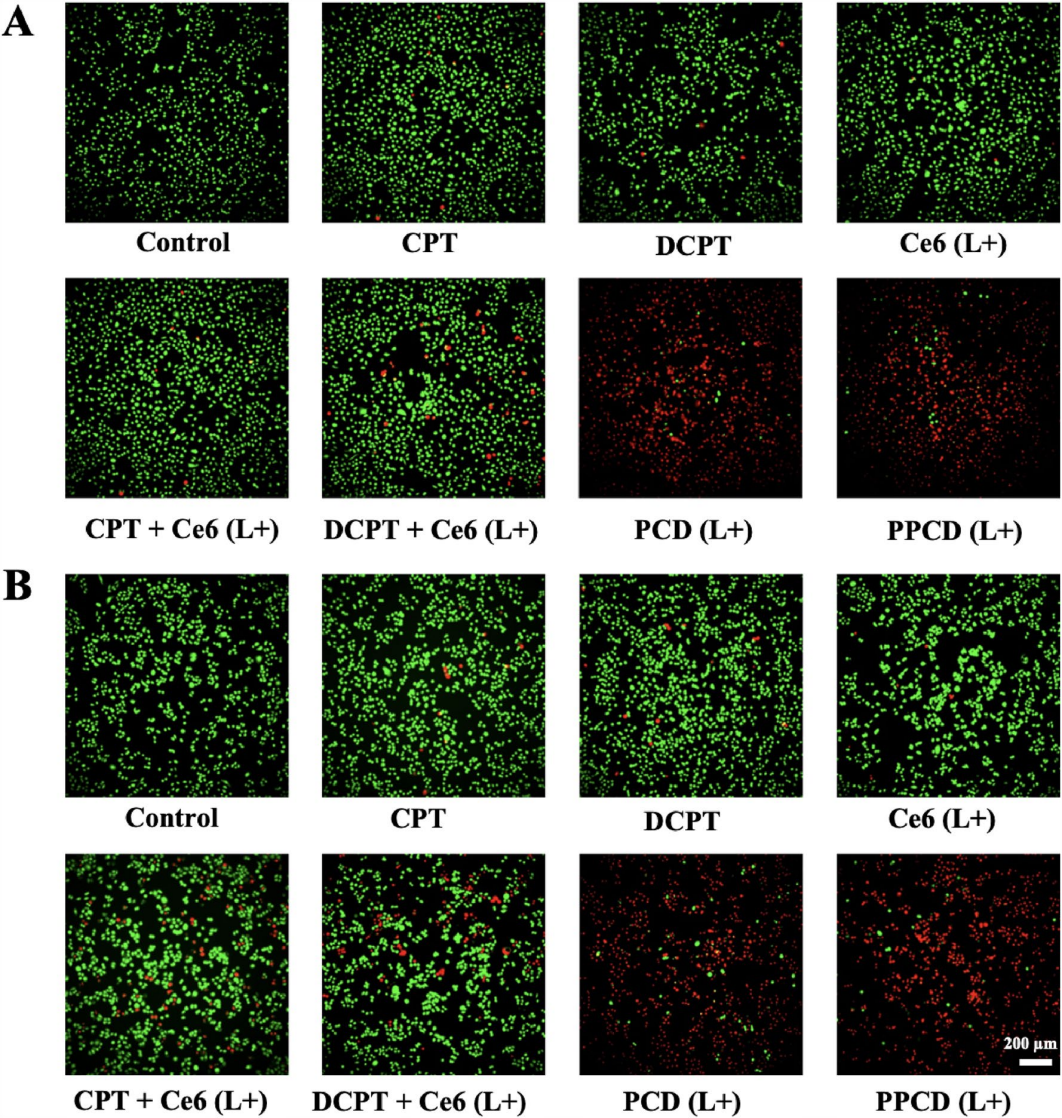
Chemo-photodynamic effects evaluation of CPT and Ce6 formulations (with laser irradiation (L+)) against A549 **A** and PC-9 **B** cells. Cells were stained with calcein-AM and PI. The scale bar represents 200 μm

In addition, calcein AM/PI was utilized to label the cells for direct observation to assess the chemo-photodynamic efficacy. In Fig. 7, viable cells were stained with green fluorescence, while non-viable cells were visualized in red. After treating the cells with PCD or PPCD micelles and subsequent irradiation with a 660 nm laser, cells exhibited striking red fluorescence signals, indicating the strong anti-cancer effect of micelle-based chemo-photodynamic combination therapy. These observations showed the same trends when compared to the results obtained in MTT assay. It should be noticed that, acting as an anti-cancer agent, CPT can kill the cancer cells when a certain drug concentration is reached. In this study, despite the fact that only low concentration of CPT or Ce6 (with laser) was employed so that the cell killing effect of single therapeutic agent was not strong, the combination medication system showed efficient cell killing ability in such concentrations, which provided evidence to the excellent anti-cancer activities of PCD and PPCD in chemo-photodynamic combination therapy.

### Cell apoptosis

**Fig. 8:**
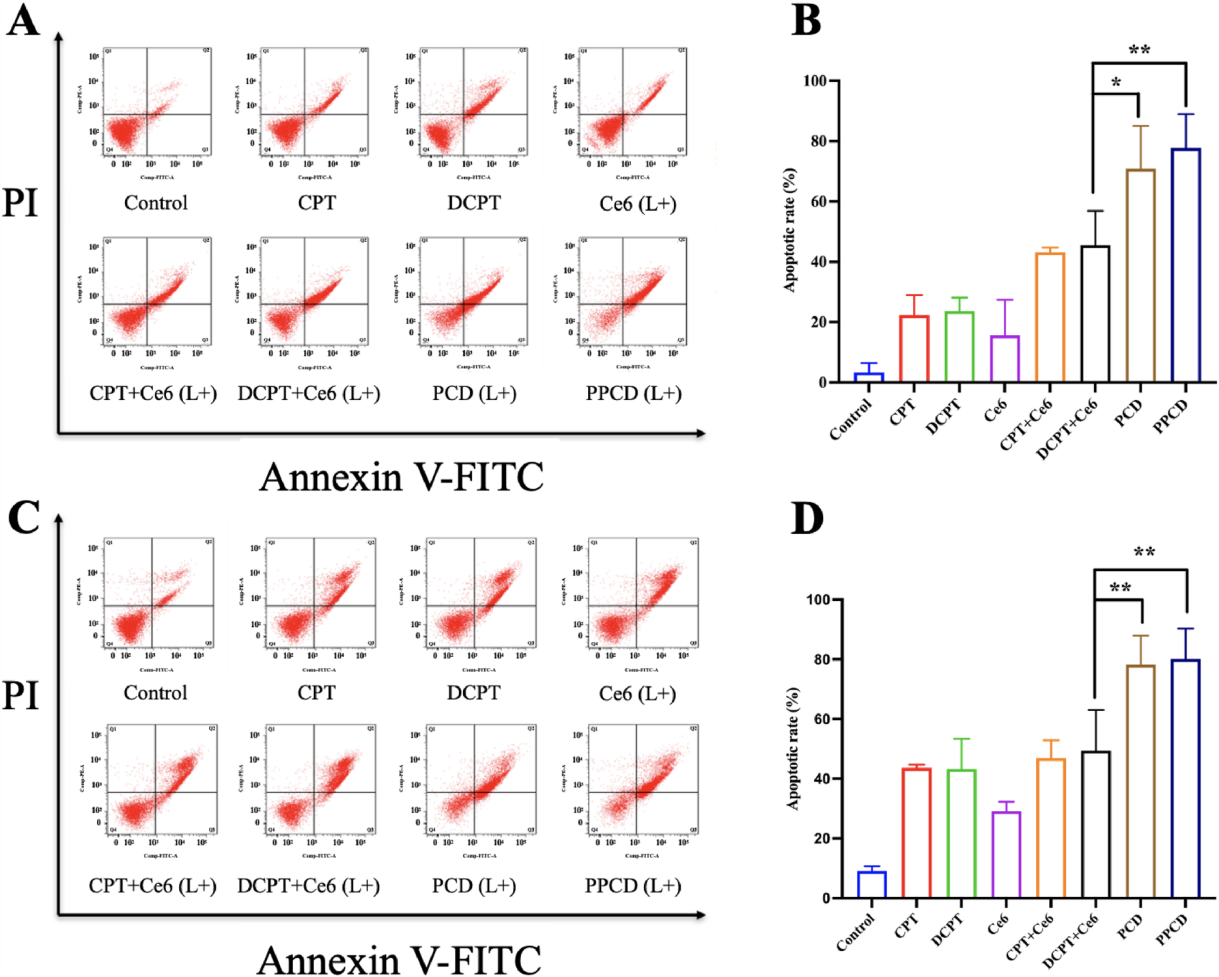
Cell apoptosis in A549 **A** and PC-9 **C** cells after treating with CPT and Ce6 formulations (with laser irradiation (L+)). The apoptosis rate **B**, **D** was the combined value of the early apoptosis rate (Q4) and the late apoptosis rate (Q2) through calculation

Annexin V-FITC/PI staining was further performed to study apoptosis in lung cancer cell lines A549 and PC-9 (Fig. 8) by flow cytometry. Apparently, slight apoptosis was induced by free CPT, DCPT or Ce6. And CPT or DCPT plus Ce6 groups exhibited moderate apoptotic cell death. PCD and PPCD micelles caused maximal apoptotic rates, which were over 70% in A549 cells and close to 80% in PC-9 cells. The results were consistent with MTT results.

### Pharmacokinetics, in vivo therapeutic efficacy, and histological assessment

**Fig. 9:**
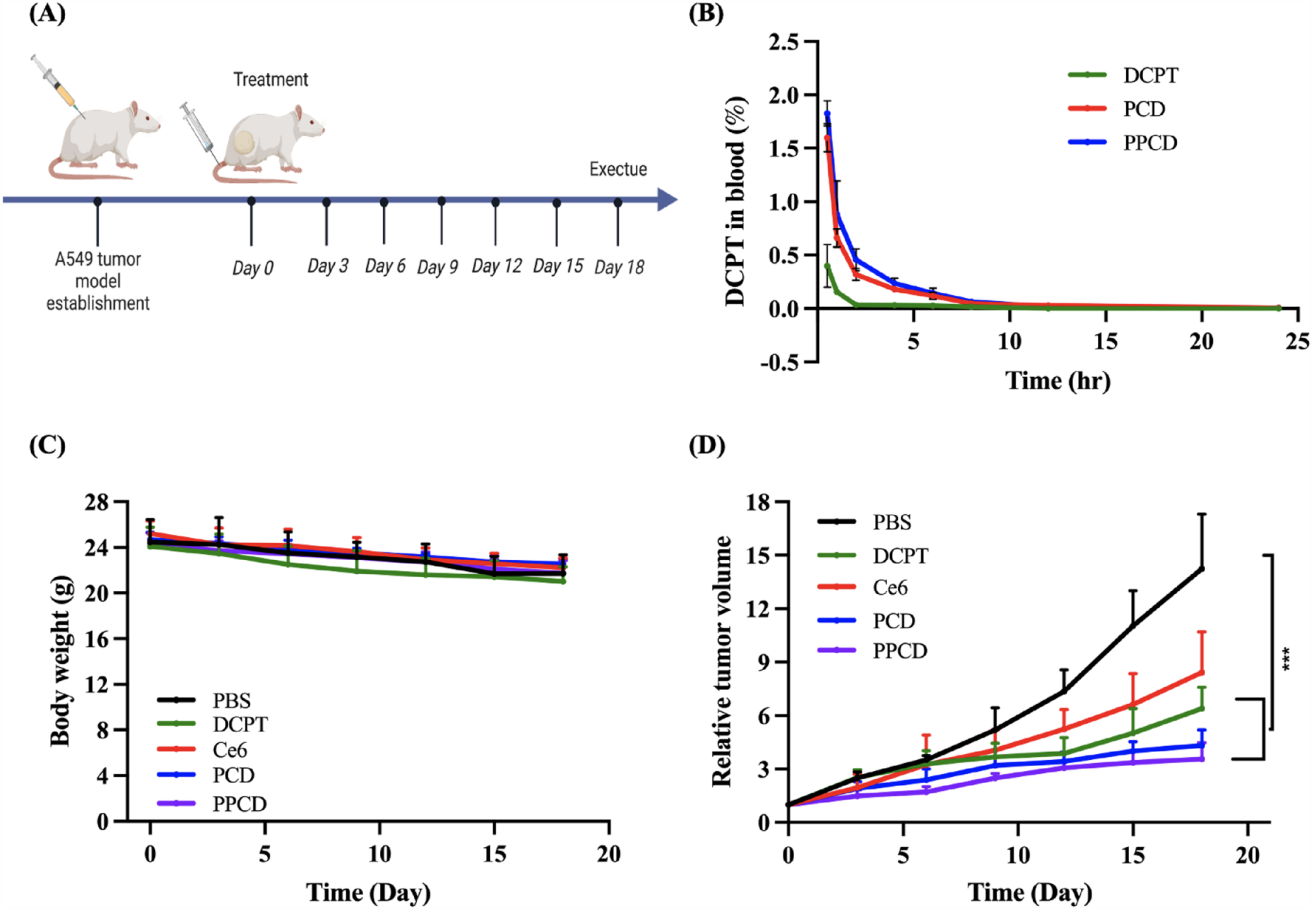
Evaluation of in vivo therapy. **A** Timeline of antitumor therapy in A549 tumor-bearing mice model; **B** Pharmacokinetics profiles of DCPT, PCD and PPCD after administration in mice (n = 3); **C** average body weight curves of mice subjected to differents treatment (n = 5); **D** relative tumor volume growing trend with various treatments (n = 5)

According to Fig. 9B and Additional file 1: Table S2, it can be seen that the concentration of DCPT in the bloodstream notably increased when encapsulated within the micelles, which was consistent to the previous report [29]. The increase should be due to the improvement of DCPT solubility and bioavailablity. In addition, the DCPT concentration in blood after administration of PPCD was a bit higher than that of PCD, which was attributed to the existance of CHO-PEG-CHO. Similar trend was observed when determining the pharmacokinetic profiles of Ce6 (Additional file 1: Fig. S2 and Table S3). Results indicated that the bioavailablity of DCPT was improved after encapsulation in micelles.

When evaluating the *in vio* therapeutic efficacy, the trend of tumor growth was obviously inhibited by all formulations except the control group (Fig. 9D). The tumors of PBS group grew rapidly while PPCD treatment exhibited the strongest tumor suppressing ability, showing the advantages of chemo-photodynamic combination therapy as compared to photodynamic theray (Ce6) and chemodyanmic therapy (DPCT). In Fig. 9C, the body weight of mice experienced a slight decline over the treatment in all groups, which was also observed in previous publication [18]. Nevertherless, no damage on H&E-stained slices of collected organs was observed, indicating that the formulated treatments did not induce singificant toxicity to the mice (Additional file 1: Fig. S3).

## Conclusions

In conclude, a dual-responsive DCPT/Ce6 co-delivery system (PPCD) was constructed for efficient chemo-photodynamic combination cancer therapy. Decoration of pH-sensitive PEG layer effectively reduced the surface charge of the nanoparticles. In acidic environment (pH 6.5), the PEG shell was detached to expose the positive-charged nanoparticles, which facilitated the cellular uptake of PPCD micelles. Furthermore, upon laser irradiation, PCD and PPCD micelles generated more amount of ROS than the free drugs to kill the cancer cells, resulting in enhanced cell inhibitory effects and significant cell apoptosis. In vivo evaluation confirmed the therapeutic potential of PCD and PPCD for cancer therapy. Overall, this PKF polypeptide-based co-delivery system is an easy, reproducible and scalable nanoplatform to integrate small molecule-based anticancer agents for effective combination therapy.

### Supplementary Information

**Additional file 1: Figure S1.** Particle size and PDI of PKF-Ce6 blank micelles. **Figure S2.** Pharmacokinetics profiles of Ce6 after administration in mice (n=3). **Figure S3.** H&E staining of major organs (heart, liver, spleen, lung and kidneys) collected from one mouse after treatment, scale bar is 20 μm. **Table S1.** Particle size of PCD micelles with different ratio of Ce6 to DCPT. **Table S2.** Pharmacokinetic parameters of DCPT, PCD and PPCD after administration in mice (n=3). **Table S3.** Pharmacokinetic parameters of Ce6, PCD and PPCD after administration in mice (n=3).

## Data Availability

All the data of this study are available from the corresponding author upon reasonable request.
