# Supplementary material for: Co-delivery of dimeric camptothecin and chlorin e6 via polypeptide-based micelles for chemo-photodynamic synergistic therapy
Source: Chin Med. 2023 Oct 13;18:133. doi: 10.1186/s13020-023-00817-6 (PMC10576266; doi:10.1186/s13020-023-00817-6)
Supplement: Supplementary file 1 — Additional file 1: Figure S1. Particle size and PDI of PKF-Ce6 blank micelles. Figure S2. Pharmacokinetics profiles of Ce6 after administration in mice (n=3). Figure S3. H&E staining of major organs (heart, liver, spleen, lung and kidneys) collected from one mouse after treatment, scale bar is 20 μm. Table S1. Particle size of PCD micelles with different ratio of Ce6 to DCPT. Table S2. Pharmacokinetic parameters of DCPT, PCD and PPCD after administration in mice (n=3). Table S3. Pharmacokinetic parameters of Ce6, PCD and PPCD after administration in mice (n=3). [file 13020_2023_817_MOESM1_ESM.docx]

**Supporting Information**

**Co-delivery of dimeric camptothecin and chlorin e6 via polypeptide-based micelles for chemo-photodynamic synergistic therapy**

Zhaopei Guo ^a^, Ka Hong Wong ^a^, Enze Li ^a^, Xingzhi Zhou ^a^, Di Jiang ^a^, Jiebing Gao ^b*^, Meiwan Chen ^a*^

^a^State Key Laboratory of Quality Research in Chinese Medicine, Institute of Chinese Medical Sciences, University of Macau, Macau SAR, China

^b^Department of Radiology, The Fifth Affiliated Hospital, Sun Yat-sen University, Zhuhai, 519000, China

*Corresponding author: [mwchen@um.edu.mo](mailto:mwchen@um.edu.mo) (M. Chen), [gaojb@mail.sysu.edu.cn](mailto:gaojb@mail.sysu.edu.cn) (J. Gao)


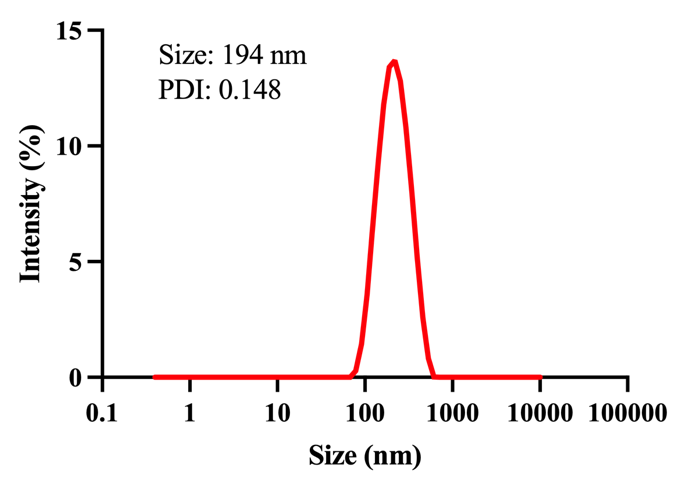


Fig. S1. Particle size and PDI of PKF-Ce6 blank micelles.


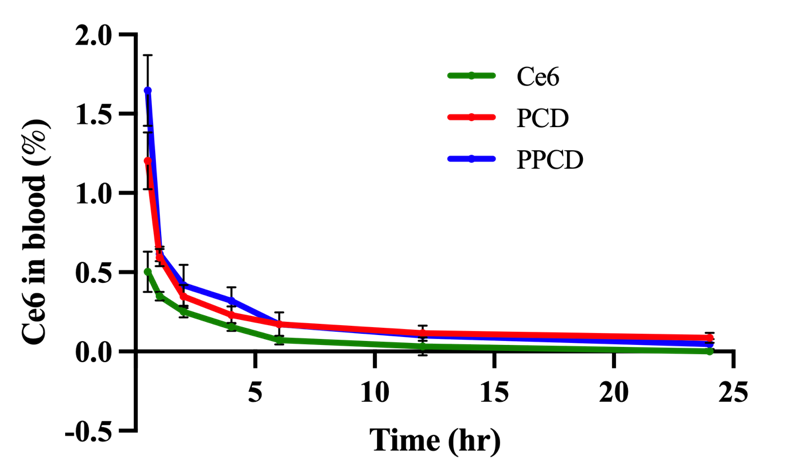


Fig. S2. Pharmacokinetics profiles of Ce6 after administration in mice (n=3)


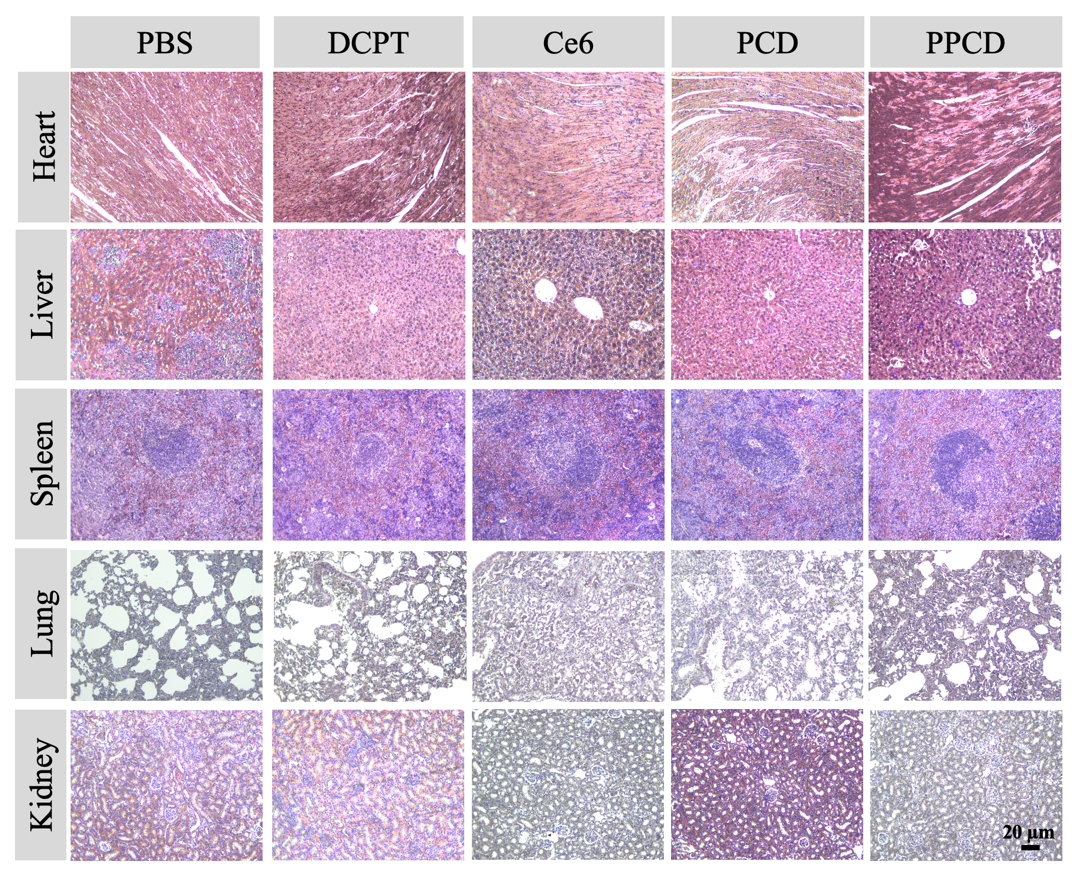


Fig. S3. H&E staining of major organs (heart, liver, spleen, lung and kidneys) collected from one mouse after treatment, scale bar is 20 μm.

Table S1. Particle size of PCD micelles with different ratio of Ce6 to DCPT

| Ratio of Ce6 to DCPT | Particle size |
| --- | --- |
| 1:1 | 554 nm |
| 1:3 | 369 nm |
| 1:5 | 218 nm |
| 1:10 | 341 nm |

Table S2. Pharmacokinetic parameters of DCPT, PCD and PPCD after administration in mice (n=3)

|  | **t_1/2_ (h)** | **AUC_(0-t)_** |
| --- | --- | --- |
| **DCPT** | 1.950 ± 0.027 | 0.956 ± 0.086 |
| **PCD** | 3.652 ± 0.154 | 2.243 ± 0.024 |
| **PPCD** | 2.205 ± 0.261 | 2.650 ± 0.254 |

Table S3. Pharmacokinetic parameters of Ce6, PCD and PPCD after administration in mice (n=3)

|  | **t_1/2_ (h)** | **AUC_(0-t)_** |
| --- | --- | --- |
| **Ce6** | 1.187 ± 0.242 | 0.489 ± 0.223 |
| **PCD** | 3.505 ± 0.804 | 2.350 ± 0.197 |
| **PPCD** | 2.898 ± 0.439 | 2.737 ± 0.073 |
